# Psoriasis: Classical vs. Paradoxical. The Yin-Yang of TNF and Type I Interferon

**DOI:** 10.3389/fimmu.2018.02746

**Published:** 2018-11-28

**Authors:** Alessio Mylonas, Curdin Conrad

**Affiliations:** Department of Dermatology, University Hospital CHUV, Lausanne, Switzerland

**Keywords:** plaque psoriasis, paradoxical psoriasis, TNF, IL-23, T_H_17, type I-interferon

## Abstract

Chronic plaque psoriasis is a common debilitating skin disease. The identification of the pathogenic role of the TNF/IL-23/T_H_17 pathway has enabled the development of targeted therapies used in the clinic today. Particularly, TNF inhibitors have become a benchmark for the treatment of numerous chronic inflammatory diseases such as psoriasis. Although being highly effective in psoriasis treatment, anti-TNFs can themselves induce psoriasis-like skin lesions, a side effect called paradoxical psoriasis. In this review, we provide a comprehensive look at the different cellular and molecular players involved in classical plaque psoriasis and contrast its pathogenesis to paradoxical psoriasis, which is clinically similar but immunologically distinct. Classical psoriasis is a T-cell mediated autoimmune disease driven by TNF, characterised by T-cells memory, and a relapsing disease course. In contrast, paradoxical psoriasis is caused by the absence of TNF and represents an ongoing type-I interferon-driven innate inflammation that fails to elicit T-cell autoimmunity and lacks memory T cell-mediated relapses.

## Introduction

Psoriasis is a distinctly human, chronic, inflammatory skin disease, affecting 2–3% of the population worldwide, with prevalence varying considerably according to race and geographic location (1). Clinically, plaque type psoriasis, the most common form of psoriasis, is characterised by well-demarcated erythematous lesions covered with silvery-white scales. These lesions are histologically reflected by keratinocyte hyperproliferation leading to epidermal hyperplasia (acanthosis), characteristic elongation of the rete ridges (papillomatosis), thickening of the cornified layer (hyperkeratosis), and incomplete keratinocyte differentiation resulting in retention of nuclei in the stratum corneum (parakeratosis). Leukocytes, including T-cells, dendritic cells, neutrophils, and macrophages make up a considerable dermal and epidermal immune cell infiltrate. Psoriasis is caused by the interaction of predisposing genetic factors and environmental triggers leading to dysregulated innate and adaptive immune responses. Today, psoriasis is widely regarded as a T-cell-mediated autoimmune disease and skin infiltrating T lymphocytes play key effector roles by driving disease development and maintenance. Dendritic cells producing TNF and IL-23 stimulate activation of both CD4^+^ and CD8^+^ T-cells, which in turn migrate into the epidermis. Upon recognition of autoantigens, T-cells produce T_H_17-cytokines such as IL-17A, IL-17F, and IL-22, which drive the psoriatic phenotype by inducing keratinocyte hyperproliferation. In support of this, several single nucleotide polymorphisms cluster throughout this pathway including genes in the TNF/dendritic cell activation pathway (*TNFAIP3, REL, TN1P1, NFKBIA*) as well as in the T-cell activation (*HLA-Cw6, ERAP1/ZAP70, ETS1, SOCS1, TNFRSF9*), and T_H_17/T_C_17-differentiation pathways (*IL23A, IL23R*) (2, 3). Consequently, antibodies targeting the pathogenic TNF/IL-23/IL-17 pathway have revolutionised psoriasis treatment over the past 15 years and are widely used in the clinic today.

In particular, TNF blockade has become the benchmark in management of numerous chronic inflammatory diseases, such as rheumatoid arthritis, Crohn's disease, and psoriasis (4–7). As such, more than two million patients have already been treated with anti-TNFs and, with the advent of biosimilars, these figures are expected to grow further over the coming years. Yet, targeting TNF is not without consequence, as TNF is a potent pro-inflammatory cytokine known to coordinate immune responses and play an important role in limiting the spread of infectious pathogens. Thus, TNF blockade leads to an increased risk of infections and slightly increased risk for certain malignancies. However, more surprisingly, anti-TNF treatment can also induce new psoriasis-like skin lesions in about 2–5% of treated patients (8).

As anti-TNFs are amongst the most potent anti-inflammatory drugs used in the treatment of psoriasis, developing psoriasis-like skin lesions due to TNF blockade was somewhat paradoxical—hence the designation “paradoxical psoriasis.”

This review aims to provide a focussed overview of the latest developments in the T-cell and cytokine networks in classical psoriasis, and contrast them to paradoxical psoriasis induced by anti-TNFs, which is clinically similar to psoriasis but immunologically distinct. Finally, these findings will be put into perspective with future avenues of research and possible clinical interventions.

## Classical psoriasis

### The established role of T-helper and the revisited cytotoxic T-cells

The pathogenic role for T-cells in psoriasis is well-established and stems from the following clinical observations and experimental findings: Immunosuppressive agents, such as cyclosporine, or therapies specifically targeting T-cells are efficacious in psoriasis treatment (9–12). *HLA-Cw6* represents the strongest genetic risk variant associated with psoriasis (13). Molecular analysis of psoriasis tissue showed that lesional T-cells are oligoclonal (14) and recognise epidermal autoantigens (15–18). Finally, clinically relevant xenotransplant models of psoriasis have demonstrated an essential functional role for T-cells (19–21).

T-cells migrate into inflamed skin through expression of the skin-homing Cutaneous Lymphocyte-associated Antigen (CLA) (22), LFA-1 and α_4_β_1_ (23), and the chemokine receptors CCR8 and CCR10 (24). More specifically T_H_1 cells use CXCR3 and CCR4 (25), whereas T_H_17 cells use CCR4 and CCR6 (26). Among the most well-described chemokines involved in T-cell migration to the skin are CCL27 (27, 28), and CCL20 (29) produced by keratinocytes upon an inflammatory trigger. While circulating T-cells certainly play an important role in skin immunopathology, there are twice as many T-cells residing in normal healthy skin than are present in the circulation (22). Moreover, pathogenic oligoclonal T-cells remain resident in resolved psoriatic skin lesions suggesting that disease recurrence might be initiated through reactivation of skin-resident T-cells (30). Indeed, these skin-resident memory T-cells were found to be sufficient to drive psoriasis development without further recruitment of circulating cells (19, 20). Activation within the skin led to proliferation of T-cells in the dermal compartment, which preceded keratinocyte hyperproliferation. In fact, the psoriatic phenotype was only induced by migration of T-cells into the epidermis and blockade of the epidermal infiltration by T-cells prevented the development of a psoriatic lesion (20). These findings suggest that intraepidermal T-cells reflect key effector cells in psoriasis.

Traditionally, much attention has been given to differentiated CD4^+^ T-cell subsets across chronic inflammatory diseases (31–34), including psoriasis (35). However, CD8^+^ T-cells, which are present in healthy skin as tissue resident memory T-cells (36), have been shown to produce a similar cytokine profile (37). In psoriasis, dermal T-cell infiltrates are mostly comprised of CD4^+^ cells, whereas the majority of T-cells in the epidermis—which represent key effector cells—are CD8^+^ (19). Indeed, we could recently show that intraepidermal CD8^+^ T-cells are functionally essential for psoriasis (38).

Psoriasis has been studied extensively from a genetics perspective, with HLA class I alleles known for more than 40 years to be heavily implicated (39). The *HLA-Cw6* variant is the strongest psoriasis susceptibility allele and has 10-fold higher association with early-onset severe psoriasis. As to how exactly class I HLA molecules might contribute to the pathogenesis of psoriasis is not entirely clear. But in light of the fundamental role of epidermal CD8^+^ T-cells in psoriasis, the fact that lesional T-cells are of oligoclonal origin and CD8^+^ T-cells recognise peptide antigens presented on MHC class I molecules suggest a role for epidermal (auto-)antigens in psoriasis. As mentioned above, epidermal CD8^+^ T-cells in psoriasis are key effectors in psoriasis (20), and they are of oligoclonal origin (14, 30)—thus potentially recognising common antigens. Taken together with *HLA-Cw6* representing the strongest genetic risk variant associated with psoriasis, this suggests that recognition of epidermal (auto-)antigens by CD8^+^ T-cells is pathogenic in psoriasis.

Indeed, the streptococcal M protein from *Streptococcus pyogenes* has been identified as an antigen target of primarily CD8^+^ T-cells (40). T-cells directed against the streptococcal M-protein had the ability to react to keratin 14, which is overexpressed in psoriatic skin, due to sequence homology and antigenic similarity (molecular mimicry). Thus, the immune response to a streptococcal infection could divert T-cells toward skin antigens and cause skin pathology. Intriguingly, streptococcal throat infections are a well-known trigger factor for onset and exacerbation of psoriasis.

Other recently identified epidermal autoantigens include keratin 7 (41) and the antimicrobial peptide LL37 expressed by keratinocytes (17) as well as the melanocyte antigen ADAMTSL5 (18). Finally, CD1a-restricted lipids were also found to elicit T-cell responses in psoriatic patients (42). Interestingly, CD1a-autoreactive T-cells isolated from skin were identified as T_H_22 cells producing IL-22 (43), a cytokine overexpressed in psoriasis and known to drive keratinocyte hyperproliferation.

Antigen-recognition by T-cells is thought to play a pivotal role in psoriasis, but an all-encompassing consensus on the nature of autoreactivity has yet to be reached. Despite this, all of the identified auto-antigens to date are significantly upregulated in psoriatic skin as compared to uninvolved or healthy skin. Because the majority can be induced locally upon injury, the prevailing model postulates that skin trauma could lead to upregulation of putative auto-antigens and their recognition by tissue-resident antigen-experienced T-cells in psoriasis patients.

### Cytokine networks: the TNF/IL-23/IL-17 axis

Nowadays, the pathogenic role of the TNF/IL-23/T_H_17 axis in psoriasis is well-known and numerous biologics targeting the different cytokines of this pro-inflammatory pathway are widely used in the clinic. Yet, the arrival of TNF blockers in the early 2000s completely revolutionised the management of psoriasis and other chronic inflammatory diseases. Despite underwhelming results of anti-TNF in sepsis (44), the successful use in rheumatoid arthritis (RA) spurred trials in other chronic inflammatory diseases such as Crohn's disease, psoriasis and psoriatic arthritis (45).

TNF is known to be potently produced by immune and non-immune cells including macrophages, T-cells, dendritic cells (DC), neutrophils, and fibroblasts. One of its major roles is to mount appropriate adaptive immune responses to tumors and pathogens. This is achieved through several mechanisms. Induction of DC-maturation leads to upregulation of CD40, CD80, CD83, and CD86 thereby potentiating T-cell receptor (TCR)-mediated responses and amplifying weak antigen affinity interactions (46). It also serves to limit the immune-suppressive effects of regulatory T-cells (47) and to enhance proliferation and survival of committed effector memory T-cells. In line with these findings, TNF is critically required to mount effective CD8^+^ T-cell responses against tumors and for the recruitment of T-cells into tumor sites (48). These pro-inflammatory effects of TNF are corroborated in psoriasis, where TNF is found to dictate the inflammatory environment in several ways (49–52). In detailed histological and molecular investigations, it was found to be mostly produced by mature conventional DCs. Blockade of TNF leads to an initial reduction of the chemokine CCL20, which preferentially recruits T_H_17 cells into inflamed tissue, coinciding with loss of IL-17 and diminution of dermal and epidermal T-cells. In addition, it leads to normalisation of DC numbers and reduction of IL-23 cytokine expression, followed by normalised keratinocyte differentiation, and eventually to histological improvement and clinical response. Taken together, TNF maintains a pro-inflammatory environment that primes pathogenic T_H_17 T-cells through induction of IL-23, maintaining them at the site of inflammation, and sustaining T_H_17 cytokine production (53, 54).

Though TNF might contribute to increased IL-17 production by T_H_17 cells (55), IL-23 directly governs T_H_17 cytokine production both by critically participating in T_H_17 cell polarisation as well as by stimulating production of IL-17 by differentiated T_H_17 cells (56, 57). Initial supportive evidence for a functional role of IL-23 in psoriasis included the clinical efficacy of an anti-p40 monoclonal antibody (blocking both IL-12 and IL-23) in psoriasis (58) and the association of a single nucleotide polymorphism in the *IL23R* gene in psoriasis patients (55, 59). Confirmation soon followed by the successful use of IL-23-specific antibodies in clinically relevant mouse models and then in patients (60, 61). In addition, IL-4 abrogated T_H_17 cell-mediated inflammation by selectively silencing IL-23 in antigen-presenting cells while sparing IL-12/T_H_1 immunity (62) and resulting in therapeutic outcome. In psoriasis, IL-23 is mainly produced by activated DCs but keratinocytes and other non-immune cells probably contribute to its production. In fact, it has been shown recently that TNF-dependent epigenetic control of IL-23 expression in keratinocytes plays a role in chronic skin inflammation (63).

T_H_17 cytokines, such as IL-17A, IL-17F, and IL-22, represent the key effector cytokines in psoriasis pathogenesis as they directly drive the development of a psoriatic phenotype. They induce epidermal hyperproliferation, attract neutrophils to the skin, and activate keratinocytes to produce chemokines and antimicrobial peptides, which sustain the inflammatory process (64). There are six homologous IL-17 cytokines (A through F) which are produced by either haematopoietic or non-haematopoietic cells, can signal through different combinations of receptors, and mediate distinct biological activities. The individual members are reviewed in this issue by Brembilla et al. (65). Besides the aforementioned effects in the pathogenesis of psoriasis, IL-17 can act in synergy with TNF to further potentiate expression of multiple pro-inflammatory mediators known to play a role in psoriasis, such as IL-8, beta-defensins, S100A proteins, IL-19, and CCL20 (29, 66–68). As such, concurrent inhibition of TNF and IL17 might result in more effective therapy. Though, a bi-specific dual variable domain immunoglobulin targeting both cytokines did not demonstrate increased efficacy compared to anti-TNF in RA (69), this remains to be tested in psoriasis.

While antibodies targeting IL-17 have shown great efficacy in psoriasis (53, 70), blockade of IL-22 failed to meet primary end points in clinical trials indicating distinct role for IL-17 and IL-22 in psoriasis. IL-22, which is produced by T_H_17 cells (71) and exclusively by a distinct T_H_22 subpopulation (72, 73), had previously been regarded as a very promising target. In psoriasis, IL-22 is found to be produced by dermal CD4^+^ but also by epidermal CD8^+^ T_C_17 and CD4^+^ T_H_22 cells (38, 74, 75), as well as Innate Lymphoid Cells (76, 77), and mast cells (78). Interestingly, skin-resident T-cells mediating disease memory in clinically resolved psoriasis plaques were found to be epidermal T_C_17 and T_H_22 cells producing mainly IL-22 (75). Upon binding to its heterodimeric receptor consisting of IL-22RA1 and IL10Rβ (79), which is expressed on keratinocytes (80), IL-22 induces proliferation of keratinocytes and inhibits terminal maturation (81, 82). While transient expression of IL-22 upon skin injury promotes epidermal remodelling with re-epithelisation of skin wounds (74), its chronic expression by psoriatic T-cells drives keratinocyte hyperproliferation and epidermal hyperplasia. In line with this, in transgenic mice, IL-22 was sufficient to induce a skin phenotype that resembles psoriasis (82), and the psoriatic phenotype induced by skin injection of IL-23 was abrogated in IL-22-deficient mice (83). However, the pathogenic function of IL-22 shows redundancy with other members of the IL-20 subfamily of cytokines such as IL-19 and IL-20, potentially rendering its blockade clinically ineffective in humans.

**Figure 1:**
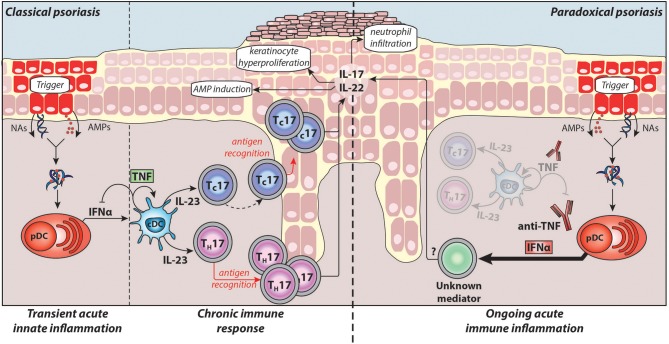
Pathogenesis of classical plaque psoriasis and paradoxical psoriasis. Antimicrobial peptides (AMPs), which are produced by keratinocytes upon skin injury or released by neutrophils, form complexes with nucleic acids (NAs) released by dying cells. These complexes activate plasmacytoid dendritic cells (pDC) to produce large amounts IFNα during the acute/early phase of psoriasis pathogenesis. IFNα activates conventional dendritic cells (cDCs), which in turn produce TNF and IL-23. TNF induces the maturation of cDCs and pDCs, which lose their ability to produce IFNα. Thus, in classical psoriasis, early IFNα production gets relayed by TNF that controls and limits the IFNα production by pDCs via a negative feedback loop (through induction of pDC maturation). Subsequently, IL-23 and other pro-inflammatory cytokines produced by cDCs drive the activation of potentially autoreactive T-cells, which proliferate and, particularly CD8^+^ T_C_ cells, migrate into the epidermis. Upon antigen recognition they produce the T_H_17 cytokines IL-17 and IL-22 that induce keratinocyte hyperproliferation, attract neutrophils to the skin, and upregulate AMP production providing a positive feedback loop eventually resulting in the psoriatic phenotype (chronic/late phase). Normally, during anti-TNF therapy, the absence of TNF and consequently of downstream cytokines suppresses pathogenic T-cells thereby alleviating classical psoriasis. However, in patients developing paradoxical psoriasis, TNF blockade inhibits pDC maturation and leads to sustained IFNα production. In addition, as cDC cannot mature in absence of TNF, paradoxical psoriasis fails to elicit a T cell mediated autoimmune response. Thus, paradoxical psoriasis remains in an ongoing IFNα-driven acute immune inflammation independent of T-cells. The exact pathogenic downstream mechanism of IFNα-driven paradoxical psoriasis skin lesions remains to be fully elucidated.

Detailed knowledge about the pathogenesis of chronic plaque psoriasis and the central role for the TNF/IL-23/T_H_17 pathway has led to the development of therapies targeting the pathogenic cytokines, including anti-TNFs, anti-p40 (IL-12/IL-23), anti-p19 (IL-23 specific), anti-IL-17A, and anti-IL-17 receptor antibodies. This pathway and its pathogenic mechanisms in classical plaque psoriasis are illustrated in Figure 1. However, less is known about instigators of psoriasis and pathogenic upstream triggers of acute cutaneous inflammation.

### Type I interferons, setting the tone for autoimmunity?

Type I IFNs are key cytokines in antiviral host defence due to their ability to limit viral replication and to induce an effective antiviral immune response (84, 85). They promote maturation of myeloid DCs and priming of CD8^+^ T-cells (86), induce T-cell proliferation (87), and sustain their survival (88). In addition, type-I IFNs stimulate differentiation of B-cells into antibody-secreting plasma cells (89). Thus, type-I IFNs are essential for the induction of an effective immune response against viruses. Although produced by all nucleated cells, they are preferentially expressed by a rare type of circulating cells called plasmacytoid dendritic cells (pDCs) (90). Upon viral recognition through endosomal Toll-like receptors (TLR) 7 and 9, pDCs produce extraordinary amounts of type-I IFN, and, therefore, have also been called professional IFN producing cells (90, 91). Under normal conditions, pDCs are not present in peripheral tissues, but they get recruited to the skin in case of infection, injury, autoimmunity, and cancer (64). Skin wounding induces rapid skin infiltration of pDCs and transient expression of type-I IFNs, which accelerate re-epithelialisation (92).

As self-nucleic acids are abundantly released into the extracellular environment during apoptotic and necrotic cell death, it is essential that pDCs avoid inappropriate activation by host-derived nucleic acid, but retain the ability to quickly respond to viral DNA and RNA. To achieve this, TLRs that sense nucleic acids are located intracellularly within endosomes, which prevents activation by extracellular self-DNA/RNA but allows immune response to viruses that actively invade the cells. Moreover, extracellular nucleases rapidly degrade nucleic acid released by dying cells without affecting DNA or RNA contained within viruses (93). Thus, under normal circumstances host-derived self-DNA/RNA released by apoptotic or damaged cells cannot activate TLR9 and 7. However, they can become potent triggers of pDC activation and type-I IFN production in the presence of endogenous antimicrobial peptides (AMP) such as LL37 and beta-defensins (94–96). AMPs are typically not expressed in healthy skin under steady-state conditions, but are transiently produced by keratinocytes or released by infiltrating neutrophils in response to skin wounding or infections (97–99). Their cationic and amphipathic structure allows AMPs to interact with and disrupt microbial membranes, which typically contain a high degree of negative charges (100). Besides their role as direct effector molecules against microorganisms, AMPs are also involved in the initiation of inflammation by breaking innate tolerance to otherwise inert extracellular self-DNA and self-RNA. Cationic AMPs bind to negatively charged fragments of nucleic acid to form aggregated and condensed structures that are resistant to extracellular degradation. Translocation of these complexes into endosomes and activation of TLR7 and 8 (RNA), or TLR9 (DNA) lead to sustained production of IFNα and IFNβ by pDCs (94–96).

Under physiological conditions, AMP-expression with activation of pDCs by AMP-nucleic acid complexes is transient, controlled by the damaging or infectious stimulus. By contrast, in psoriasis, the expression of AMPs is persistent and leads to sustained production of type-I IFNs by pDCs, which accumulate in the dermis of early developing psoriatic lesions (101). Subsequently, these trigger activation of myeloid DCs and autoreactive T-cells. Recent work has demonstrated that IFNα particularly drives the activation and skin infiltration of pathogenic CD8^+^ T-cells in psoriasis (102). Moreover, IFNα conditioned DCs produce large amounts of IL-23 (103, 104) indicating an important role for type-I IFNs in driving T_H_/T_C_17-mediated (auto)immunity in psoriasis. Indeed, depletion of pDCs or blocking type-I IFN signalling both inhibited psoriasis development confirming that its overexpression by pDCs reflects a critical early/acute event in the pathogenesis of psoriasis (101). This is further supported by the observations that *de novo* psoriasis or pre-existing psoriasis can be triggered and/or aggravated by IFNα therapy (105–108) and the TLR7 agonist imiquimod (109, 110). Interestingly, epidermal trauma, which induces AMP expression by keratinocytes and attracts pDCs into the skin, is also a typical trigger of psoriasis known as Koebner phenomenon.

Taken together, type-I IFNs play a critical role in the acute/early phase of psoriasis pathogenesis by (1) activating dermal myeloid DC, (2) inducing their maturation by upregulating co-stimulatory molecules and HLA molecules, and (3) participating in T_H_/T_C_17 polarisation of autoimmune T-cells through induction of IL-23 production by myeloid DCs (Figure 1).

## Paradoxical psoriasis

Almost two decades of clinical experience with anti-TNFs have provided considerable advances in our understanding of the biology of TNF. More than 2 million patients have been treated with anti-TNFs so far.

Expected side effects such as increased susceptibility to infection and a slightly increased risk for malignancies have been confirmed (111–113), though the cancer risk still remains a matter of debate (114, 115). However, the observation that anti-TNFs, which are normally extremely effective in the treatment of chronic inflammatory diseases, could lead to aggravation of pre-existing autoimmune diseases and onset of new inflammatory diseases, was unexpected and a paradox. In fact, lupus-like syndrome can be observed in 0.5–1% of anti-TNF treated patients and 2–5% of patients develop psoriasis-like skin lesions, called paradoxical psoriasis (8, 116, 117). They represent important side effects in the treatment of major chronic autoimmune diseases as they potentially necessitate treatment cessation. Since the first description of paradoxical psoriasis (117, 118), numerous cases have been reported (119–121). Paradoxical psoriasis appears independently of the underlying disease or the type of anti-TNF agent used and regresses upon discontinuation of therapy, which suggests that paradoxical psoriasis does represent a side effect of TNF blockade and not *de novo* psoriasis. Though the side effect is a well-established phenomenon, its pathogenesis had remained elusive and only recently, the dysbalance of TNF and type-I IFN (yin-yang of TNF and IFNα) has been confirmed as a pathogenic mechanism underlying paradoxical psoriasis (8).

The first clues for a link between anti-TNF therapy and increased type-I IFN expression came from the observation that anti-TNF therapy induces an IFN signature in blood of juvenile arthritis patients (122). Likewise, anti-TNF treatments promote formation of anti-nuclear antibodies (123), which are associated with increased type-I IFN levels in SLE patients (124). Furthermore, anti-TNFs can induce or aggravate lupus, a well-known type-I IFN-driven autoimmune disease (125, 126). Indeed, patients with anti-TNF induced paradoxical psoriasis showed an increased IFN signature in lesional skin (127). Recently, we could confirm that TNF controls the production of type I-IFN by pDCs and that anti-TNF induces its unabated overexpression driving paradoxical psoriasis (8).

Upon activation, pDCs produce type-I IFNs first, which is relayed by their production of TNF. TNF induces maturation of pDCs, which upregulate costimulatory molecules and lose their ability to produce interferons (8, 128). Thereby, TNF limits the duration of type-I IFN production by pDCs, while conversely, TNF blockade decreases pDC maturation and extends their ability to produce type-I IFN. This supports a yin-yang model of TNF and type-I IFN. In classical plaque psoriasis, early transient overexpression of type-I IFN is replaced by a dominant TNF-driven chronic inflammation. In contrast, TNF blockade leads to an ongoing type-I IFN mediated acute inflammation in paradoxical psoriasis (Figure 1).

Another important distinction between the two entities is that in paradoxical psoriasis, unlike classical psoriasis, T-cells play a redundant role (8). Hence, both classical and paradoxical psoriasis are induced by pDC-derived type-I IFN. But while classical psoriasis develops into a T-cell mediated autoimmune disease, paradoxical psoriasis represents an ongoing type-I IFN-driven innate immune response that fails to elicit T cell autoimmunity. In line with this, there are no relapses of paradoxical psoriasis upon discontinuation of anti-TNF therapy, which supports lack of T-cell mediated disease memory in paradoxical psoriasis.

It remains unclear what triggers the activation of pDCs and eventually drives paradoxical psoriasis. Potentially certain environmental factors such as microbes could trigger development of paradoxical psoriasis, as a considerable number of patients have been found to have concurrent superinfections (129). Furthermore, the yin-yang of TNF and type-I IFN, the pathogenic mechanism underlying paradoxical psoriasis, is inherently true for healthy individuals as much as patients. But only 2–5% of anti-TNF treated patients develop paradoxical psoriasis indicating that there is another key determining factor such as genetic predisposition for paradoxical psoriasis. Among polymorphisms associated with psoriasis, five have been identified to also be associated to paradoxical psoriasis, and these include *IL23R, FBXL19, CTLA4, SLC12A8*, and *TAP1* (130) though it remains to be determined exactly how they would fit in the pathological mechanism.

Though classical and paradoxical psoriasis have similarities in their clinical presentation, many distinctions have been identified in recent years as to the pathogenic mechanism. Table 1 summarises key similarities and differences between the two entities, and highlights potential treatment strategies.

**Table 1:** Classical vs. Paradoxical psoriasis- differences, similarities, and treatment strategies.

**Characteristics**	**Classical psoriasis**	**Paradoxical psoriasis**
Clinico-phenotypic presentation	Well-demarcated erythematous plaques covered with silvery-white scales.	Presence of different psoriatic patterns including plaque-type, guttate, pustular forms as well as eczematiform presentation. Palmoplantar zones affected more often. Non-cicatricial alopecia regularly noted.
Histo-pathological appearance	Characteristic psoriatic histology: Epidermal hyperplasia (acanthosis), papillomatosis, hyper-/parakeratosis, dermal, and epidermal immune cell infiltrates.	Three different patterns: -classical psoriatic pattern -eczematiform pattern with spongiosis -lichenoid pattern with interface dermatitis often all these patterns are simultaneously present at variable degrees.
Recurrence	Relapsing.	Non-relapsing (upon cessation of anti-TNF).
Genetic associations	Many known (and established): *HLA-Cw6, IL12B, IL23A, IL23R*, and various components along type-I interferon signalling, NF-KB signalling, and other signalling pathways.	Few proposed: *IL23R* (an allele that is protective concerning classical psoriasis), and *FBXL19, CTLA4, SCL12A8, TAPl* which have an unclear role in paradoxical psoriasis and the outcome of the allele is undetermined.
Role of TNF	Driven by TNF.	Induced by blockade of TNF.
Role of adaptive immunity	T-cell mediated. Intraepidermal and dermal (autoimmune) T_H_/T_C_17-cells found throughout skin lesions.	T-cell independent. Significant reduced numbers of intraepidermal CD8^+^ T_C_-cells as compared to classical psoriasis.
Role of innate immunity	Transiently driven by pDC derived type-I IFN during the early phase of psoriasis development. Mature cDCs and neutrophils present in large numbers in skin lesions of chronic/late phase of classical psoriasis.	Driven by unabated type-I IFN produced by non-maturing pDCs. Immature dendritic cells, and neutrophils often present in lesions. Role for other cell types not known (particularly in mediating the psoriatic phenotype).
Pathogenic mechanism	Chronic (autoimmune) T_H_/T_C_17-mediated inflammation	Unabated, ongoing type-I IFN-driven innate inflammation, absence of T-cell autoimmunity.
Treatment avenues	-targeting TNF highly effective	-switch to different class of biologics (other than anti-TNF) often needed in severe cases of paradoxical psoriasis
	Various other treatment strategies validated:	In the absence of detailed knowledge about the pathogenic pathways, proposition of:
	-targeting of IL-12/IL-23 highly effective -targeting of IL-23 highly effective -targeting IL-17A and its receptor highly effective -targeting type-I interferon is ineffective in established classical chronic plaque-type psoriasis.	-use of anti-IL12/IL23 (successful in case reports) -unknown efficacy of IL-23 specific biologics -unknown efficacy of targeting IL-17A and its receptor -targeting type-I interferons and/or pDCs potentially effective

## Outlook

Detailed knowledge on classical plaque psoriasis, particularly by identifying the relevant role of the TNF/IL-23/T_H_17 axis in its pathogenesis, has allowed for novel, more targeted therapies.

Though newer treatments targeting IL-23 and IL-17 show better efficacy, today, anti-TNFs remain a gold-standard in psoriasis management. Yet important immunological side effects of TNF blockade, such as paradoxical psoriasis and lupus-like syndrome, may require premature discontinuation of an otherwise effective treatment option for patients. These side effects are caused by an unabated type-I IFN-driven immune response making it an intriguing target in these patients. While, anti-IFNs have not shown efficacy in chronic plaque psoriasis confirming the distinct inflammatory pathways in chronic and acute forms of classical psoriasis (131), they provided promising results in SLE. Therefore, type-I IFN blockade might be a valuable treatment option in acute forms of psoriasis such as erythrodermic or guttate psoriasis as well as in paradoxical psoriasis. However, simultaneous inhibition of the interferon pathway together with the ongoing TNF blockade might increase the infectious risk too considerably. Therefore, targeting IFN-producing pDCs (i.e., *via* anti-ILT-7, anti-BDCA2) or inhibiting TLR 7 and 9, thereby blocking pDC activation, could provide more suitable therapeutic options in patients that need continuation of their anti-TNF treatment. In this way, production of type-I IFNs by monocytes and stromal cells would remain intact and might allow sufficient immune responses toward infectious agents.

Despite considerable advances in the understanding of paradoxical psoriasis and its pathogenesis, several questions are still unanswered. Downstream mechanisms that mediate the interferon-driven psoriatic phenotype of paradoxical psoriasis remain unknown as IFNα does not directly induce keratinocyte hyperproliferation. The identification of cytokines involved and their cellular source might provide additional novel targets for therapeutic intervention. In addition, biomarkers to predict side effects such as paradoxical psoriasis and lupus-like syndrome could help optimising the management of patients with chronic inflammatory diseases.

## Author contributions

AM and CC wrote and edited the manuscript and figures.

### Conflict of interest statement

The authors declare that the research was conducted in the absence of any commercial or financial relationships that could be construed as a potential conflict of interest.
